# Ethnic inequality within the elderly population in utilizing healthcare services

**DOI:** 10.1186/s13584-019-0311-y

**Published:** 2019-05-01

**Authors:** B. Adini

**Affiliations:** Department of Emergency Management & Disaster Medicine, School of Public Health, Sackler Faculty of Medicine, Tel Aviv University, Tel Aviv-Jaffa, Israel

**Keywords:** Minority, Inequality, Elderly population, Ethnicity, Utilization of healthcare, Health literacy

## Abstract

The accessibility of minority ethnic groups to healthcare services is challenging in many societies, most especially among the elderly population. Elderly individuals from minority groups have been found to have lower levels of utilizing healthcare services, including preventive care, intensive hospital care, advanced technological procedures and rehabilitation. Universal health coverage is incapable of addressing all of healthcare’s access inequities and there is a need to assess the overall outcomes, including mortality rates over time, functionality of discharged patients, quality of life and/or unplanned readmissions that may indicate low quality hospital discharge processes. There is a need to investigate the impact of perceived trust/distrust in the healthcare system of elderly patients from minority ethnicities on their willingness to consume medical services.

To ensure equity in service provision, there is a need to examine whether medical providers, even unconsciously, prioritize vital services, such as rehabilitation services to populations that share similar social backgrounds. An essential measure is enhancement of health literacy at all levels, from the individual to policy-makers and strategic adoption of health literacy programs that encompass all ethnicities, considering their respective needs, norms and expectations.

Ethnic equality in accessing medical services is crucial in view of the numerous migrants and asylum-seekers who look for refuge in varied societies globally. Such populations are perceived as faring worse in healthcare quality of care, and this highlights the need to adapt the healthcare systems to the varied health behaviors, contextual factors, language barriers, lower health literacy levels and limited access to timely care. Improving equity and access to medical care is dependent on enhanced health literacy; policies that consider diverse needs of majority and minority groups; and advanced research. Concurrent implementation of these measures will be well aligned with the global strive to promote the Sustainable Development Goals (SDGs).

## Ethnic inequality within the elderly population in utilizing healthcare services

In their paper concerning ethnic equality between elderly Jewish versus Arab casualties in utilizing the Israeli trauma healthcare system, Abdel-Rahman et al. (2019) [1] highlight existing disparities between the two populations, most specifically concerning pre- and post-hospitalization services. The authors should be commended for highlighting this important issue, as information on inequity in providing medical services at large and specifically to the vulnerable elderly population, needs to be presented in order to motivate action to decrease inequality in the consumption of healthcare services.

Accessibility of patients from minority ethnic groups to healthcare services has been recognized as a challenge in many societies world-wide, and such inequality has been especially identified among the elderly population [2–4]. Walton & Anthony (2017) [5] found that while Latino, Black and Native American elderly individuals utilized less medical services than white peers, they expressed higher needs for utilization rates of medical services, even when other factors such as health needs, socio-economic levels or the medical systems’ features are accounted for. Similar minority ethnic groups also have a lower access to preventive medical services, intensive hospital care and advanced technological procedures [6, 7].

As all Israeli residents are entitled to medical services based on the National Health Insurance Law, it is not surprising that no significant differences were found between the two ethnic groups concerning utilization of surgical procedures and mortality during the term of hospitalization. As previously stipulated, universal health coverage endeavors to contribute towards quality of care [8]. Medical services within hospitals are provided based on the needs that are identified by each institution’s medical personnel and consequently, similar utilization of inpatient care is expected. Nonetheless, universal health coverage is incapable of adequately addressing all of healthcare’s access inequities. Therefore, there is a need to assess the overall outcomes of the two ethnic populations, including mortality rates over time (not just within the specific hospitalization period following the trauma), functionality of the discharged patients, quality of life and/or unplanned readmissions that may indicate low quality hospital discharge processes [7, 9].

Considering the shorter length of stays that was found concerning elderly Arab versus Jewish patients, assessing recurrent readmissions may be an important indicator for quality of care [10].

Abdel-Rahman et al. (2019) [1] note that though there is a difference in levels of accessibility of the elderly Arab population to vital inpatient hospital services such as intensive care units, the diversity is even more notable concerning pre-hospital evacuation from home to hospital by professional ambulance services and post-hospital rehabilitation services. Access to high-quality rehabilitation programs, a service that is frequently in shortage and characterized as a bottle-neck, is found to be significantly lower among elderly minority groups compared to the majority population in many countries [11, 12]. Inequality in utilization of rehabilitation services and barriers in accessing such programs may result from varied causes, such as budgetary considerations, distrust in the system, social contexts (caring for the elderly at home as a core value of family cohesiveness and respect), or innate prioritization of stronger populations by healthcare providers [13, 14].

There is a need to investigate the impact of perceived trust/distrust in the healthcare system of the elderly patients and/or their family members from minority ethnicities on their willingness to consume medical services. Distrust was presented in previous studies as a major barrier to accessing vital medical services, especially among older adults belonging to minority groups [15–17]. Trauma casualties cannot in most cases avoid medical treatment in the acute-care settings, but mistrust coupled with language barriers may cause minority patients to strive for a shorter period of stay in the hospital, accounting, at least partially, for the shorter lengths of stay and the lower access to rehabilitation programs [9]. As language is a basic component of communication and trust, the use of different mother-tongue languages may represent different social backgrounds that could lead to varied inclinations to utilize services. Even those elderly individuals from minority ethnic groups that fluently speak the language used by the majority may feel detached and unwilling to use more than the minimally essential services. To ensure equity in service provision, there is also a need to examine whether medical providers, even unconscientiously, may prioritize such services to populations that share similar social backgrounds [18].

An essential measure in overcoming this challenge is to enhance health literacy at five levels: individual, family, organizational, community and policy-making [7, 18]. This entails strategic adoption of health literacy programs that encompass all ethnicities, considering their respective needs, norms and expectations.

Abdul-Rachman et al. [1] note that some of the variance in health care utilization may be derived from the difference in urban versus periphery residence of Arab and Jewish populations. It is well recognized that populations that reside in the periphery and/or rural areas have a lower accessibility to medical services. While this has already been identified, there is merit to investigate potential diversity in utilization within both majority and minority social groups that reside in the same periphery locations. This may facilitate understanding of the internal diversity between the two ethnicities in regard to health perceptions, social conceptualities, help-seeking decisions and healthcare utilization behaviors [19].

Ethnic equality in accessing medical services becomes more urgent and crucial in view of the numerous migrants and asylum-seekers who are looking for refuge in varied societies globally. Such populations are perceived as faring worse in healthcare quality of care [9]. European and other countries are experiencing increased ethnic diversity resulting from the influx of migrants and refugees from African and Asian countries, that enhances the need to adapt the healthcare systems to the varied health behaviors, contextual factors, language barriers, lower health literacy levels and limited access to timely care [14, 15].

Improving access to medical care and equity in utilizing hospital services as well as pre and post-hospital care is dependent on multiple facets including: enhanced health education and literacy among all societal sectors; formulation of public policies that take into account the diverse needs of the population’s majority and minority groups; continuous evaluation of emerging needs and design of potential solutions through the conduct of advanced research activities [20]. Research and evaluation of service provision [21] to all societal sectors, most especially to vulnerable populations including elderly minority groups, should be continuously applied to improve efficiency of the healthcare system, eradicate barriers to accessible medical care and enhance equity in provision of services. Concurrent implementation of these objectives and measures will be well aligned with the global strive to promote the Sustainable Development Goals (SDGs) and facilitate the achievement of better health outcomes and higher social value [22, 23].

## Conclusions

Reduction of health inequalities is defined as one of the strategic aims of the Israeli Ministry of Health, as part of the universal health coverage and the overall objective to achieve a just and sustainable society. To enhance this goal, a designated “Reduction of Health Inequalities Unit” was established, responsible for planning policies and coordinating activities targeted to cultivate the capacity to fight inequity [24]. Despite the varied measures that were implemented to achieve such equity, ethnic inequalities in utilization of healthcare services still exist among the elderly population, especially in regard to pre-hospital care (evacuation to hospitals by ambulance) and post-hospitalization rehabilitation services. An important step towards reducing such inequities is a better understanding of the roots of the ethnic inequity – whether they stem from social contexts, budgetary constraints, distrust of the healthcare system, or prioritization of other populations that in essence may result in a lower accessibility to vital services. Recognition of such causes will facilitate the capacity to manage personal, organizational and societal factors that impede on the accessibility to healthcare services, and thus to achieve equity in healthcare provision.

## Abbreviation

SDGs: Sustainable Development Goals

## References

[1] Abdel-Rahman N, Yoffe N, Siman-Tov M, Radomislensky I, Peleg K (2019). Achieving ethnic equality in the Israel trauma healthcare system: the case of the elderly population. Israel journal of health policy research.

[2] Kutateladze BL, Lawson VZ (2017). A new look at inequality: introducing and testing a cross-sectional equality measurement framework in new York City. Soc Indic Res.

[3] Wang X, Yang H, Duan Z, Pan J (2018). Spatial accessibility of primary health care in China: a case study in Sichuan Province. Soc Sci Med.

[4] Sibai AM, Rizk A, Chemaitelly H (2017). Self-rated health disparities among disadvantaged older adults in ethnically diverse urban neighborhoods in a middle eastern country. Ethnicity & health.

[5] Walton E, Anthony DL. Do you want to see a doctor for that? Contextualizing racial and ethnic differences in care-seeking. InHealth and Health care concerns among women and racial and ethnic minorities 2017 Aug 10 (pp. 249-278). Emerald Publishing Limited.

[6] Fiscella K, Franks P, Gold MR, Clancy CM (2000). Inequality in quality: addressing socioeconomic, racial, and ethnic disparities in health care. Jama..

[7] Willging CE, Sommerfeld DH, Jaramillo ET, Lujan E, Bly RS, Debenport EK, Verney SP, Lujan R (2018). Improving native American elder access to and use of health care through effective health system navigation. BMC Health Serv Res.

[8] O'Connell T, K Rasanathan M (2014). ChopraWhat does universal health coverage mean?. Lancet.

[9] Katikireddi SV, Cezard G, Bhopal RS, Williams L, Douglas A, Millard A, Steiner M, Buchanan D, Sheikh A, Gruer L (2018). Assessment of health care, hospital admissions, and mortality by ethnicity: population-based cohort study of health-system performance in Scotland. Lancet Public Health.

[10] Chin DL, Bang H, Manickam RN, Romano PS (2016). Rethinking thirty-day hospital readmissions: shorter intervals might be better indicators of quality of care. Health Aff.

[11] Brzoska P, Voigtländer S, Spallek J, Razum O (2010). Utilization and effectiveness of medical rehabilitation in foreign nationals residing in Germany. Eur J Epidemiol.

[12] Kristiansen M, Razum O, Tezcan-Güntekin H, Krasnik A (2016). Aging and health among migrants in a European perspective. Public Health Rev.

[13] Frederiksen HW. Cardiac rehabilitation among migrants(doctoral dissertation, FACULTY OF HEALTH AND MEDICAL SCIENCES PhD thesis Hanne Winther Frederiksen cardiac rehabilitation among migrants-a mixed-methods study Department of Internal Medicine, Copenhagen University hospital). 2018.

[14] Yeo Y (2017). Healthcare inequality issues among immigrant elders after neoliberal welfare reform: empirical findings from the United States. Eur J Health Econ.

[15] Kristiansen M, Kessing LL, Norredam M, Krasnik A (2015). Migrants’ perceptions of aging in Denmark and attitudes towards remigration: findings from a qualitative study. BMC Health Serv Res.

[16] Jervelund SS, Maltesen T, Wimmelmann CL, Petersen JH, Krasnik A (2017). Ignorance is not bliss: the effect of systematic information on immigrants’ knowledge of and satisfaction with the Danish healthcare system. Scand J Public Health.

[17] Srivarathan A, Jensen AN, Kristiansen M (2019). Community-based interventions to enhance healthy aging in disadvantaged areas: perceptions of older adults and health care professionals. BMC Health Serv Res.

[18] Fiscella K, Sanders MR (2016). Racial and ethnic disparities in the quality of health care. Annu Rev Public Health.

[19] Heaney AK, Winter SJ (2016). Climate-driven migration: an exploratory case study of Maasai health perceptions and help-seeking behaviors. International journal of public health.

[20] Clark LA, Boyd AS (2017). Health Disparities and Social determinants of Health among the elderly. J Cult Divers.

[21] Hicken MT, Kravitz-Wirtz N, Durkee M, Jackson JS (2018). Racial inequalities in health: framing future research. Soc Sci Med.

[22] Hashemi G, Kuper H, Wickenden M. SDGs, inclusive health and the path to universal health coverage. 2017.

[23] Kruk ME, Gage AD, Arsenault C, Jordan K, Leslie HH, Roder-DeWan S, Adeyi O, Barker P, Daelmans B, Doubova SV, English M (2018). High-quality health systems in the sustainable development goals era: time for a revolution. Lancet Glob Health.

[24] Ministry of Health website. Available at: https://www.health.gov.il/English/Topics/Equality_in_Health/Pages/default.aspx . Accessed at April 16, 2019.

